# BioLogical: a universal analysis framework for biosystem logical dynamics

**DOI:** 10.1016/j.csbj.2025.11.049

**Published:** 2025-12-06

**Authors:** Yuxiang Yao, Dong Liu, Zheting Zhang, Chengchen Zhao, Duanqing Pei

**Affiliations:** a Laboratory of Cell Fate Control, School of Life Sciences, Westlake University, Hangzhou, 310030, China; b Westlake Laboratory of Life Sciences and Biomedicine, Hangzhou, 310024, China; c Key Laboratory of Biomedical Intelligent Computing Technology of Zhejiang Province, Hangzhou, China

**Keywords:** Logical analysis, Complex biosystems, Multi-valued logic, Discrete dynamics, Gene regulatory networks

## Abstract

Complex biosystems exhibit ordered, functional, self-organized features, yet a universal framework for exploring their logical paradigms and dynamic behaviors remains lacking. Here we present BioLogical, a user-friendly R package designed to analyze logical properties of gene regulatory systems. Through a standard workflow and multiple cases, we demonstrate its versatile capabilities in deciphering logical paradigms, computing static and dynamic biosystem indicators, simulating system evolution, and evaluating logical satisfiability. All concepts and algorithms are extended to multi-valued scenarios, and hierarchical interfaces are provided to meet diverse requirements. The open-source software is available at https://github.com/YuxiangYao/BioLogical.

## 1 Introduction

Biosystems often exhibit strong resistance to noise and remarkable adaptability to environmental changes, primarily due to the well-orchestrated interactions among their components. In gene regulatory networks (GRNs), these interactions require not only specific circuit topologies but also crucial modes of action [1], [2], namely intrinsic logical paradigms underlying biological processes. These paradigms, traditionally abstracted as Boolean functions [3], reveal key GRN properties absent in random systems, such as orderliness, decoupling, criticality, redundancy, and controllability [4], [5], [6], [7], [8], [9], [10], [11]. A comprehensive exploration of these logical paradigms can elucidate the fundamental mechanisms of genetic regulation, enable precise interventions in cell fate decisions, and provide a foundation for designing synthetic genetic circuits. However, existing analytical toolkits originate from diverse backgrounds and lack a universal framework or standardized pipeline for systematically investigating logical architectures and their associated dynamic behaviors. Furthermore, recent advances in sequencing technologies necessitate extending these paradigms to multi-valued scenarios, allowing for more detailed integration of multi-omics data into regulatory network modeling.

In this software article, we introduce BioLogical, a user-friendly R package designed to decode logical paradigms, calculate system order parameters, analyze logical features, perform logic conversions, and simulate discrete dynamics, offering a comprehensive toolkit for investigating the logical and dynamic properties of discrete biosystems. Moreover, BioLogical extends classical concepts and algorithms to multi-valued logic frameworks and incorporates logical satisfiability solvers, thereby overcoming the limitations of Boolean-based packages. The built-in GRN datasets, comprehensive documentation, and hierarchical function interfaces facilitate interactive exploration and support large-scale simulations. BioLogical is implemented in efficient C++ with minimal dependencies and early compatibility with R, enabling cross-platform installation through source code or precompiled binaries.

## 2 Package overview

### 2.1 System frameworks

This package focuses on the logical behaviors of complex systems and the principles governing their logical dynamics. Logical models of living systems are typically formulated within a Boolean framework, in which gene expression and silencing are represented by the values 1 and 0, respectively. The system’s dynamical behavior is determined by the logical paradigm assigned to each component, commonly expressed as Boolean functions. A Boolean function defines a mapping that specifies how the combinatorial states of a gene’s regulators determine its expression state. These functions exhibit a variety of dynamical and statistical properties, such as output stability under input perturbations, the degree of functional redundancy among upstream regulators, and variations in regulatory influence, having been extensively studied in prior work [8], [12], [13]. Together with the system’s topological structure, these logical paradigms govern the overall processes of dynamics, revealing rich emergent features in discrete systems, including criticality and scaling laws [6], [14], [15]. To address more complex modeling requirements, these concepts are extended to multi-valued systems, incorporating fractional, integer, and incomparable discrete frameworks [15], [16], [17]. The package architecture is implemented using Rcpp to ensure high computational efficiency [18], offering both standard R functions for exploratory analysis and optimized, high-performance interfaces for large-scale modeling.

To ensure cross-platform compatibility, the open-source package includes embedded dynamic libraries for Linux, Windows, and macOS, thereby eliminating dependency-related installation issues. The software is available on GitHub at https://github.com/YuxiangYao/BioLogical. Users can download platform-specific pre-compiled binaries for local installation or, more conveniently, install the package directly online using the devtools package with the command devtools::install_github (“YuxiangYao/BioLogical/pkgs/BioLogical_*/)”, where * denotes the target platform: linux for Linux, win for Windows, and mac for macOS. The package minimizes version compatibility issues by supporting widely used environments (R ⩾ 3.5, Rcpp[18] ⩾ 1.0.10). Additionally, the third-party C++ library Z3 [19], upon which BioLogical depends, has been fully integrated into the installation package.

### 2.2 Function modules

**Fig. 1:**
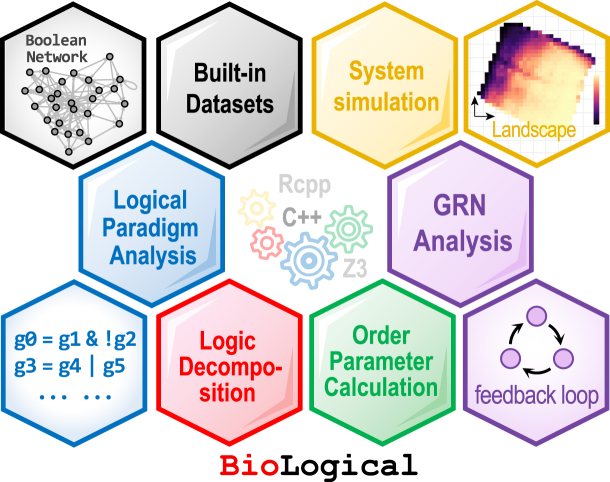
Schematic illustration of the main functional modules in BioLogical.

To address diverse analytical needs, BioLogical aggregates a variety of interrelated functional modules, organized into the following categories (Fig. 1):

**Logical paradigm analysis.** As the core functionality, BioLogical enables the identification and generation of common logical paradigms, including canalizing, signed, and dominant types, within both Boolean and multi-valued frameworks. These paradigms correspond to conditional branching, monotonic and threshold behaviors, and competitive interactions, respectively [4], [13], [20], and typically play essential roles in complex biosystems. For clarity and brevity, their detailed mathematical definitions are provided in the appendix.

**Logic decomposition and conversion.** Complex logical paradigms are composed of elementary logical operations, such as AND, OR, and NOT in Boolean systems. This module decomposes mapping relationships in both Boolean and multi-valued systems into their fundamental components, thereby revealing the underlying interactions within the system. The implementation is based on the Quine-McCluskey algorithm [21], [22]. To facilitate cross-system comparisons, the package supports logic conversion, including transitions from Boolean to multi-valued scenarios. Moreover, it provides polynomial analysis [23], which transforms logical expressions into algebraic representations to enable the study of algebraic properties such as coupling orderliness and algebraic complexity of logical paradigms.

**Order parameter calculation.** To evaluate the dynamic features of logical paradigms, the package provides three distinct order parameters: (1) *Sensitivity* measures how input perturbations affect mapping outcomes [12]; lower sensitivity values indicate greater robustness to such perturbations. (2) *Input/Edge effectiveness* quantifies the contribution of individual inputs to the output within a logical paradigm and captures the degree of redundancy among these inputs [8]. (3) *Logical complexity* estimates the complexity of a logical paradigm based on the number of its prime implicants; paradigms with higher regularity typically require fewer prime implicants for representation [24].

**Genetic network analysis.** This module focuses on investigating the logical behavior of gene regulatory networks: (1) It classifies and quantifies the *distribution of logical paradigms* within the system, such as the levels of canalization. (2) By evaluating edge validity and their transitive relationships in the network, it enables the identification of *relevant components* that characterize the system’s tendency toward stability or disorder. (3) Integrating logical paradigms with locally connected motifs, such as feed-forward loops, reveals the *core components* underlying the network’s emergent dynamical behavior. (4) Specialized functions are provided to analyze the fundamental properties of feedback loops, which are hallmark features of genetic networks.

**Built-in datasets.** The package incorporates gene regulatory network datasets from diverse sources, encompassing various species and physiological processes. These datasets serve as benchmark references and examples of biological regulatory mechanisms. To support user-defined research, the package provides multiple data input interfaces and built-in network generation functions.

**System simulation.** The package also features dynamic system simulation capabilities, such as examining how a system evolves from a random initial state to a stable state. To support theoretical research in complex systems, this module integrates two frameworks, Derrida’s damage spreading and the percolation of stable components—for analyzing the general dynamical properties of discrete systems.

## 3 A brief workflow

To clarify the package’s functionality and the underlying concepts, this section illustrates the use of BioLogical through a simple workflow. We assume the reader is familiar with the fundamental aspects of R programming. For detailed information on function usage, users are directed to the package help documentation, which provides comprehensive parameter descriptions and illustrative examples. Mathematical definitions are provided in the relevant literature and in the appendix.

### 3.1 Data input

**Fig. 2:**
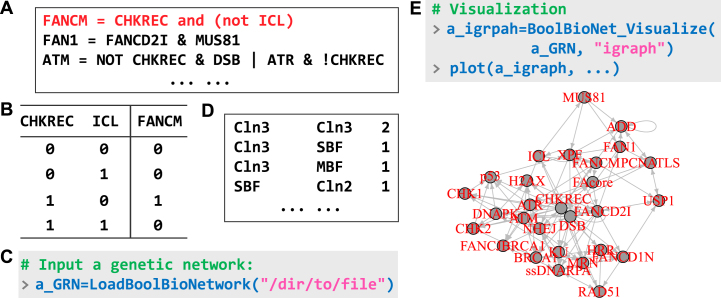
Data input. (A) Schematic illustration of a logical model input file. Each row represents the logical formula of a gene. The notation for logical operators is flexible; “AND/OR/NOT” logic can be expressed using either symbols (&, |, !) or words. (B) Truth table corresponding to the logical paradigm in the first row. Inputting networks in this format is also supported. (C) Example of the data import function, demonstrated using Cell Collective network ID 1778. In this and all subsequent figures, gray boxes represent sample code; blue, black, green, and magenta text denote R commands, return results, comments, and key control parameters, respectively. (D) Schematic illustration of a threshold model input file, exemplified by the *budding yeast cell cycle network* included in the package. The first and second columns denote the input and output genes, respectively; each row represents a regulatory interaction, with the final number specifying the edge type: 1 for activation and 2 for repression. (E) Visualization of the GRN.

The foundation of a logical model consists of logical expressions (Fig. 2A), such as “FANCM = CHKREC and (not ICL)”, indicating that FANCM is expressed only when CHKREC is active but ICL is silenced (Fig. 2B). Each gene in the model has an expression that characterizes its regulatory behavior. The package provides multiple data import interfaces for accepting complete logical expressions or corresponding truth tables of GRNs (Fig. 2C). For threshold models, a list of regulatory edges is also supported, formatted as shown in Fig. 2(D). The GRN is stored as a list comprising four sublists: gene names, input edges, output edges, and regulatory relationships (truth tables). This structured format serves as the standard input and output for all gene network analyses. Each built-in GRN is stored as an element within predefined dataset lists, BoolGRN_CellCollective, BoolGRN_KadelkaSet, and BoolGRN_ThresholdModel. Multiple export options for GRNs include conversion into input–output pairs for visualization in external software, as well as compatibility with the igraph[25] format for direct representation (Fig. 2E). BioLogical also includes a random generation function that supports diverse configurations of network structures and logical paradigms. In this section, we use several built-in networks as case studies.

### 3.2 Logical paradigm analysis

**Fig. 3:**
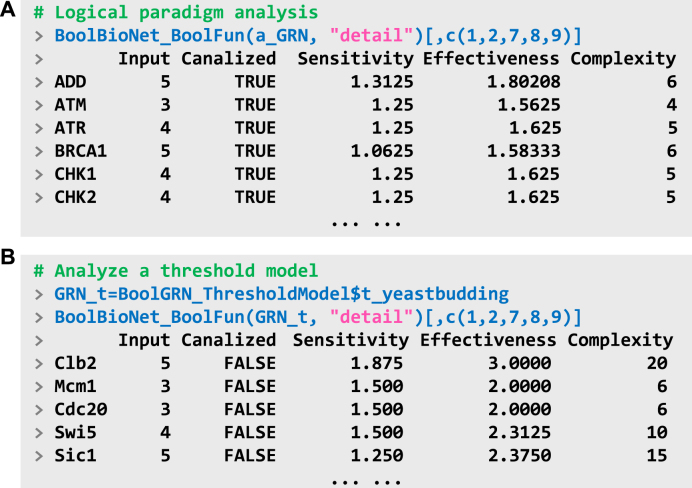
Logical paradigm analysis. (A) Analysis of the categories and properties of logical paradigms within GRNs. For clarity, only selected properties are shown; for further details, please refer to the package documentation. (B) Same analysis employing a built-in threshold model, the 11-node budding yeast cell cycle network. Note that the cases shown here are typical cases and do not imply that the canalizing property is strictly associated with the model type.

BoolBioNet_BoolFun can analyze the categories of logical paradigms for all genes in the network. As shown in the returned output (Fig. 3A), all genes exhibit canalizing regulation, indicating strongly constrained behaviors. The results also include three metrics of dynamic properties—sensitivity, input effectiveness, and logical complexity—that reflect functional characteristics such as robustness to noise and structural orderliness (see Section 3.4). The type of GRNs can be approximately inferred from the distribution of logical paradigm categories [10]. For instance, logical models typically adhere to the principle of canalization, whereas not all threshold models satisfy this property (Fig. 3B).

### 3.3 Logic decomposition and conversion

**Fig. 4:**
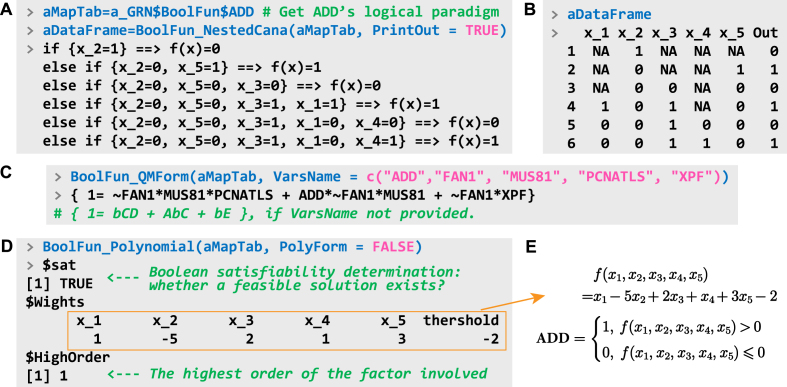
Logic decomposition and conversion of the logical paradigm of gene ADD in the case GRN. (A) Canalizing patterns. The TRUE value of the argument PrintOut indicates that the result will be printed to the terminal. “X_1, X_2, …”, denote the five inputs, “ADD, FAN1, MUS81, PCNATLS, XPF”, respectively. (B) The simplified mapping table of the logical paradigm in the format of R dataframe. (C) Obtaining the logical expression. If VarName is not provided, letters are used to denoted the variables; the lowercase letters indicate that the corresponding variable is negated. (D,E) Logical-to-threshold conversion of the logical paradigm of ADD, where $x_{1},\ldots ,x_{5}$ take values of 0 or 1.

For specific genes of interest, their regulatory behaviors can be examined in detail. The function BoolFun_NestedCana analyzes the nested canalizing relationships of a gene and directly outputs visual information in the R terminal. Fig. 4(A) provides an example of the canalizing patterns of gene ADD in the GRN “c_1778”. Clearly, variable X_2 (corresponding to the input FAN1 of ADD) plays the most crucial: when its value is 1, gene ADD is silent; by sequentially evaluating each layer, the potential expression of ADD can be determined. The function returns a simplified version of the mapping table for this canalizing pattern, where NA values indicate that the inputs at corresponding positions have no effect on the output (Fig. 4B).

Using the Quine-McCluskey method, BoolFun_QMForm can transform the logical paradigm into an expression composed of the basic logical operators. This transformation not only facilitates understanding of the collaborative relationships among upstream genes regulating ADD expression, but also reveals the source of conditional constraints (Fig. 4C). The symbols ∗, +, and ∼ denote logical operations “AND, OR, and NOT”, respectively. It can be inferred that the absence of FAN1 is a prerequisite for ADD expression. Under this condition, ADD expression is achieved through three distinct pathways: the coexistence of MUS81 and PCNATLS, the coexistence of MUS81 and ADD, or the independent activity of XPF.

Typically, even paradigms within a logic-based model may exhibit threshold-based representations. Using the Z3 solver [19], BoolFun_Polynomial can verify the existence of a threshold expression for a given logical paradigm and return a feasible solution if one exists. Fig. 4(D) shows the logical-to-threshold conversion of the logical paradigm of ADD. The function returns three key pieces of information: 1) the satisfiability of feasible solutions, 2) a possible solution, and 3) the highest order of the factor. Fig. 4(E) illustrates that the regulation of ADD expression is determined by the net effect of competitive interactions between positive and negative regulatory factors. If PolyForm is TRUE, the function returns a strict polynomial form rather than a threshold-based one. In this case, a unique solution always exists.^1^ Due to the high complexity of $Wights, only the unique solution is shown as follows:

(1) $$\begin{array}{ll} \text{ADD} & \;=x_{5}+x_{1,3}-x_{2,5}+x_{3,4}-x_{1,2,3}-x_{1,3,4}-x_{1,3,5}-x_{2,3,4}-x_{3,4,5}\\ & \;+x_{1,2,3,4}+x_{1,2,3,5}+x_{1,3,4,5}+x_{2,3,4,5}-x_{1,2,3,4,5} \end{array}$$

Where $x_{i,j,\ldots ,k}$ means “$x_{i}\times x_{j}\times \ldots \times x_{k}$”.

### 3.4 Order parameters of system dynamics

As classical indicators of dynamic properties, sensitivity (S) and edge effectiveness (E) characterize the logical paradigm in terms of perturbation resistance and input validity, respectively. S represents the sum of the expected changes in the mapping outcomes resulting from perturbing each individual input variable. A smaller S suggests greater robustness against perturbations. E represents the effectiveness of all input signals in influencing downstream outcomes and quantifies the overall redundancy of the inputs, defined as k−E, where k is the number of input variables. A smaller value of E indicates greater redundancy among input variables. In addition to BoolBioNet_BoolFun, S and E can be analyzed using specialized functions.

**Fig. 5:**
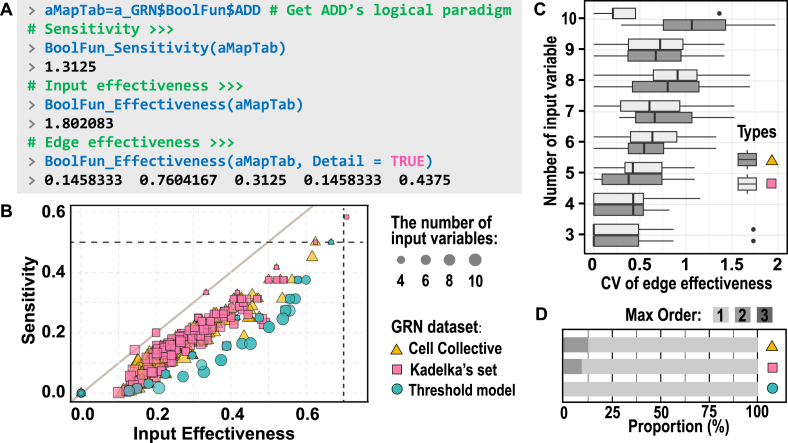
Analysis of order parameters of logical paradigms. (A) Calculation of sensitivity (S) and effectiveness (E) for the logical paradigm of ADD. Setting the argument Detail in BoolFun_Effectiveness to TRUE returns the edge effectiveness rather than the overall input one. (B) Correlation between S and E across all logical paradigms in the three GRN datasets. Both metrics are normalized by the numbers of input variable (k), with only paradigms satisfying 3⩽k⩽10 included. Dashed lines indicate random baselines for comparison; the diagonal line serves as a visual reference. (C) Coefficient of variation (CV) of edge effectiveness in logical paradigms. Logical paradigms from threshold models are excluded because their CV values are zero. (D) Proportion of highest-order terms in the threshold-form representations of logical paradigms. The symbols in panels C and D correspond to those in panel B and represent distinct datasets.

Fig. 5(A) illustrates the calculation of S and E for gene ADD. The S is 1.3125, and the overall input effectiveness $E_{\text{input}}$ is approximately 1.80. Both values are smaller than those of a random paradigm with an equivalent number of input variables (Fig. 5B), indicating the presence of robustness and redundancy in the GRNs. More specifically, the edge effectiveness values for the five input variables of ADD are as follows: ADD=0.1458, FAN1=0.7604, MUS81=0.3125, PCNATLS=0.1458, and XPF=0.4375, with their sum equaling $E_{\text{input}}$. Although these values may appear random, they partially reflect the regulatory contribution of each input variable. By comparing its logical expressions (Fig. 4C), silencing FAN1 is identified as the prerequisite step and thus exhibits the highest effectiveness. Among the three feasible pathways, XPF can activate ADD independently, whereas the other two pathways both require MUS81 in combination with either PCNATLS or ADD. Notably, the ranking of E across the five variables corresponds to the order of their absolute weights in the threshold paradigm (Fig. 4D and E), demonstrating consistency between the order parameter and the modeling framework.

To illustrate the relationship between S and E, these two normalized metrics were calculated for all genes across the three datasets, as shown in Fig. 5(B). The two metrics generally exhibit a strong positive correlation, indicating that greater input effectiveness increases the susceptibility of the logical paradigm to input perturbations. Notably, significant differences are observed across model types: the (E,S) distribution of logical paradigms in the threshold-based model is shifted downward relative to that in the purely logical models (whose paradigms are predominantly canalizing). This implies that, at equivalent effectiveness levels, threshold paradigms are more robust against perturbations.

To explain this phenomenon, we calculated the differences in edge effectiveness ($E_{\text{edge}}$) across all paradigms. The coefficient of variation (CV), defined as the ratio of the standard deviation to the mean, is used to quantify the heterogeneity of $E_{\text{edge}}$. As shown in Fig. 5(C), the $E_{\text{edge}}$ values of paradigms from the logical model exhibit greater heterogeneity and a slight increase with the number of input variables. This suggests that variations in external inputs more readily alter the mapping outcomes. From another perspective, we examined the distribution of the highest-order terms in the paradigms after conversion to threshold form. Fig. 5(D) reveals that although most paradigms are linear, some still include second-order or higher-order terms. This indicates the presence of coupling behavior among multiple variables, implying that variations in input variables may have a broader impact on the mapping outcomes.

### 3.5 System component analysis

**Fig. 6:**
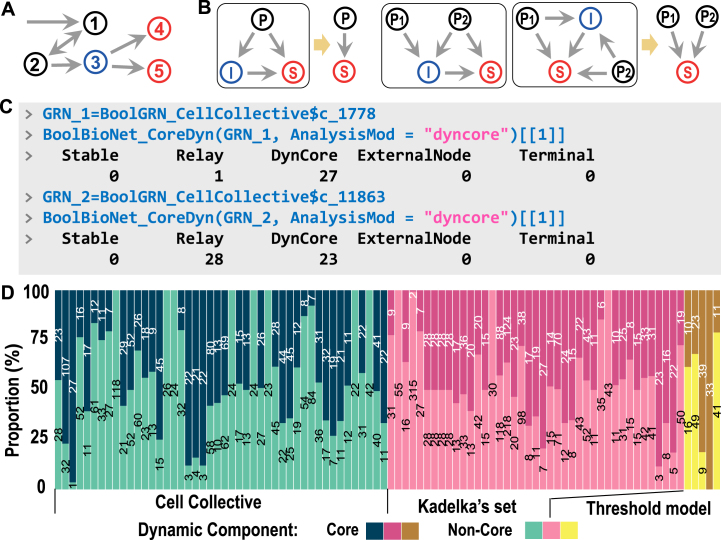
Analysis of system component. (A) Schematic diagram of the system component categories. Gene4 and Gene5 represent the terminal nodes, while Gene3 serves as a relay node, as it provides input only to these terminal nodes. None of Gene3/4/5 are relevant components. (B) Schematic illustration of FFL simplification through logical optimization, in which intermediate nodes are removed. (C) Examples of dynamic core component analysis, illustrated using Cell Collective ID 1778 and ID 11863. For simplicity, only the first element of the returned list is presented (“[[1]]”). Readers may consult the help documentation and execute the program to view other information, such as the optimized GRN. The function AnalysisMod accepts the argument “relevant” to analyze the relevant components. (D) Proportions of dynamic core and non-core components in GRNs. Only networks with system sizes greater than 30 are shown.

In GRNs, certain genes receive regulatory signals from upstream inputs and do not regulate any downstream genes; these are referred to as terminal genes. Similarly, genes whose downstream targets consist solely of terminal genes are classified as relay genes. Both exhibit limited regulatory potential, as their signals are predictable and fail to propagate throughout the entire system (Fig. 6A). The portion of the GRN that excludes terminal and relay genes is defined as the relevant components [26], which typically serve as candidate controllers of the system from a dynamic perspective. Moreover, GRNs contain numerous feedforward loops (FFLs) [1], whose logical behaviors provide an additional perspective for system simplification. As shown in Fig. 6(B), after logical satisfiability analysis, FFLs retain only the signal input nodes (predecessors/**P**s) and the output node (successor/**S**), with intermediate nodes (**I**s) removed through optimization. This refinement further narrows the candidate set of potential control factors, called dynamic core components.

Fig. 6(C) displays the analysis of core components (CCs) using BioLogical. According to the results, the components of GRN_1 (ID: c_1778) are as follows: it lacks stable genes, terminal genes, and external inputs, and only one gene serves as the relay gene. For comparison, GRN_2 (ID: c_11863) is analyzed as a representative example, revealing that nearly half of its genes can be optimized. This marked difference between the two networks arises from the topological preferences of GRNs, such as the prevalence of loop structures and the degree of edge coupling (see Section 3.6 for detailed analysis). Fig. 6(D) illustrates the heterogeneous distribution of CCs across GRNs in the three datasets. In most GRNs, the proportion of CCs is below 50 %, suggesting that the fundamental logical behavior of the system arises from a relatively small set of key genes. It is important to emphasize that CCs differ from the actual control set; rather, they capture the structural and logical interactions within GRNs.

### 3.6 Feedback loop identification

**Fig. 7:**
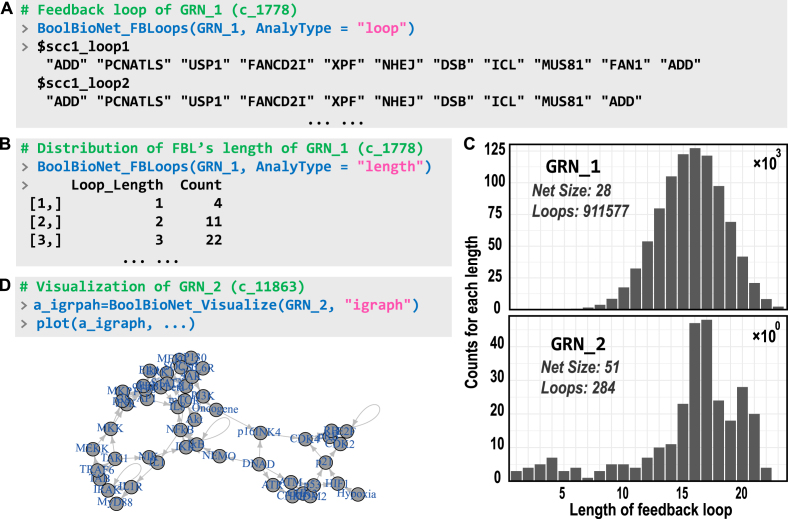
Analysis of feedback loop (FBL) in GRNs. (A,B) Examples of FBL detection and length distribution analysis. (C) Distribution of FBL lengths for the two example networks, Cell Collective IDs 1778 (GRN_1) and 11863 (GRN_2). Note the difference in the scale of the vertical axis. (D) Network visualization of GRN_2.

Feedback loops (FBLs) in GRNs directly influence the emergence of oscillatory behavior in the system [27]. BioLogical provides a dedicated function, BoolBioNet_FBLoops, for analyzing FBLs and their properties. By setting AnalyType to loop, the function returns a list containing all existing FBLs, with each element representing a complete FBL (Fig. 7A). The name of each FBL denotes which strongly connected component it belongs to. The distribution of FBL lengths within the system can be obtained by setting the parameter length (Fig. 7B). We analyzed the FBLs of two sample networks in Section 3.5 and presented their distributions. As shown in Fig. 7(C), although smaller in size than GRN_2, GRN_1 exhibits significantly more FBLs, indicating stronger coupling and greater circularity. Fig. 7(D) presents the network schematic of GRN_2, which exhibits a significantly lower coupling degree than GRN_1 (Fig. 2E).

**Fig. 8:**
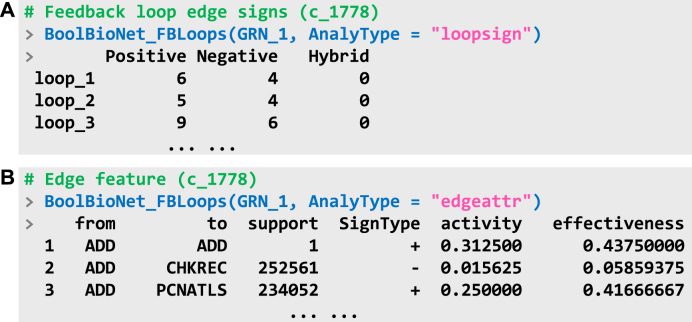
Properties of edges in feedback loops (FBLs). (A) Signs of the constituent edges of each loop. (B) Detailed information and properties of each edge within FBLs.

The signs of the regulatory edges that form these loops are also critical in determining system behaviors. A positive edge $A\overset{(+)\;}{\to }B$ is defined such that, when the inputs from other genes are held constant, the expression level of gene B under the condition A=1 is always greater than or equal to the expression level under A=0. Conversely, a negative edge ($A\overset{(-)\;}{\to }B$) is defined when the expression of B under A=1 is consistently less than or equal to its expression under A=0. If neither condition holds, the edge is classified as hybrid ($A\overset{(?)\;}{\to }B$). Fig. 8(A) presents the analysis of edge signs and the corresponding results obtained by setting the parameter AnalyType to loopsign. The results confirm that, in GRN_1, regulatory edges primarily occur as distinct positive or negative influences, with no hybrid ones observed, indicating that gene activities exhibit clear directional characteristics. By setting AnalyType to edgeattr, the function can perform a more detailed analysis of edge features across all loops, including their signs, activity levels, and effectiveness (Fig. 8B). The activity level quantifies the robustness of an individual edge to noise. The column support represents the number of FBLs in which the corresponding edge appears.

### 3.7 Dynamic simulation

**Fig. 9:**
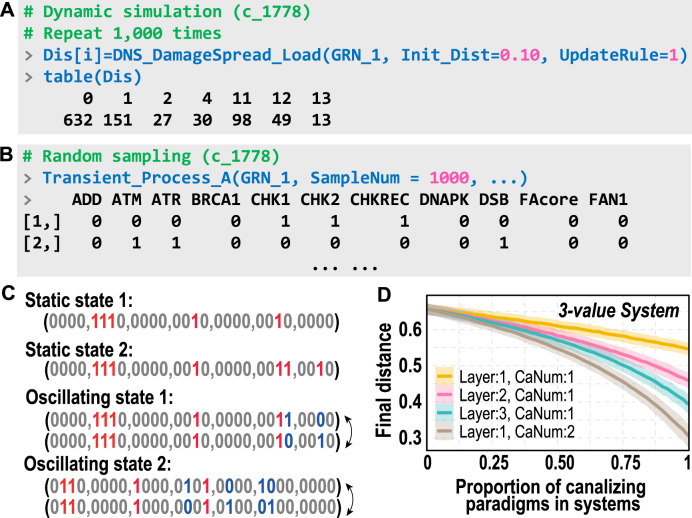
Dynamic simulation of GRNs and theoretical models. (A) Calculation process and distribution visualization of final distance differences caused by damage spread after perturbation. Init_Dist means 10% genes are perturbed. UpdateRule specifies the system dynamics update rule, where 1 for synchronous updating and 2 for asynchronous updating. Other settings retain their default values; refer to the help documentation for further details. (B) Search for possible steady states in gene regulatory GRNs through random sampling. The parameter SampleNum indicates the number of samples generated, with each line of the results representing a potential steady state. (C) Schematic illustration of the steady states in GRN_1, including two static and two oscillatory attractors. Red and blue numbers represent the non-zero static and oscillating genes, respectively. The difference between the corresponding states exactly matches the distance scenarios listed in (A). (D) Damage spread analysis of 3-value Kauffman networks (K=4, Size=1,000) incorporating canalizing paradigms (Layer, canalizing layer/depth; CanNum, number of canalizing variables). Small perturbations (10%) ultimately propagate across the entire system, as evidenced by the final distances.

Dynamic simulations reveal cell fate differentiation influenced by logical paradigms. For a system state s and its perturbed state $s^{\prime }$, the distance between them is defined as the number of components that differ between the two states. The analysis of damage spreading examines whether this distance increases or decreases after a given period of time evolution, corresponding respectively to a steady or a chaotic-like system [28]. The example network GRN_1 was simulated using DNS_DamageSpread_Load. Distances following 1,000 perturbations were computed, and their distribution is shown in Fig. 9(A). Although most distances are small (less than 4, accounting for 84%), there are still instances with relatively large distances.

By randomly initializing sampling using Transient_Process_A, six possible states were observed as the system evolved to stability or oscillation (Fig. 9B and C). They form two static states (s) and two oscillating states (o), namely $s_{1}:(x_{5,6,7,15,23}=1)$, $s_{2}:(x_{5,6,7,15,23,24,27}=1)$, $o_{1}:(x_{5,6,7,15,23;(24)}=1)↔(x_{5,6,7,15,23;(27)}=1)$, and $o_{2}:(x_{2,3,9,16;(18,22)}=1)↔(x_{2,3,9,16;(14,21)}=1)$. Only non-zero variable indices are displayed, with those in parentheses denoting oscillatory components. All such states are referred to as attractors. The well-established R package for attractor identification is currently available [29], rendering further discussion of attractor features unnecessary.

Discrete dynamical systems, as classical models of complex systems, provide a fundamental framework for studying critical and self-organized behaviors in dynamical processes. The package includes built-in tools for logical dynamical analysis, such as percolation and scaling law analyses, specifically designed for researchers in the field of complex systems. These tools facilitate investigations into phenomena such as the effect of increasing canalization on system stability within the context of the Kauffman model or other types of networks (Fig. 9D). Notably, greater depths of canalizing layers and a higher number of canalizing values are associated with increased system stability. For detailed theoretical research, readers are referred to the comprehensive package documentation.

## 4 Application cases of multi-valued systems

Multi-valued systems have already been applied in the electronic engineering field, with corresponding devices already designed [30], [31]; however, their engineering application in biological systems remains in its early stages [32]. In theoretical biology, the analysis of multi-valued behavior in biological systems is primarily at the stage of theoretical exploration and involves simple discrete models [33], [34], [35].

Recent advancements in multi-valued discrete modeling have demonstrated the practical applications of such systems. Kreuzaler et al. proposed a multi-valued model for the systematic investigation of heterogeneity in Myc expression within breast cancer [36]. Cells with high Myc expression (Myc${\;}^{\text{H}}$) exhibit proliferative capacity, whereas those with low Myc expression (Myc${\;}^{\text{L}}$) maintain Wnt signaling; collectively, these cell populations cooperate to shape the tumor microenvironment. Based on this model, Trinh et al. further analyzed the potential attractor states, identifying specific values associated with proliferation and apoptosis to evaluate intervention strategies [17]. In these cases, employing a binary Boolean system fails to capture the full range of system states.

**Fig. 10:**
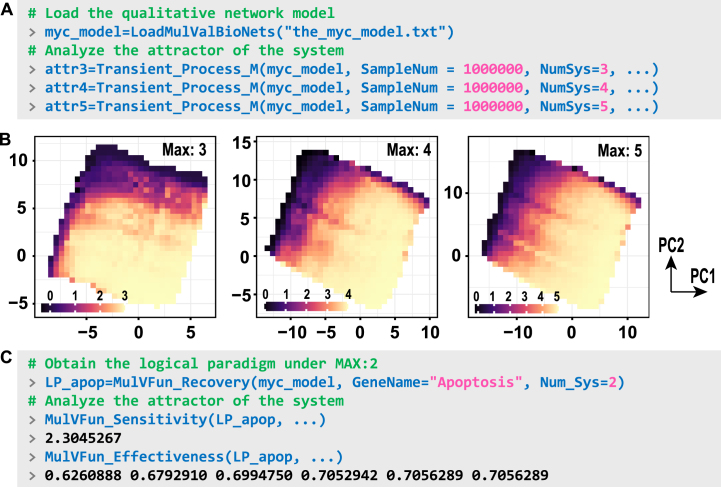
Analysis of a multi-valued network associated with breast cancer. (A) Input the network and simulate it using various allowable system maximum values. The parameter SampleNum denotes the number of random sampling. (B) Phenotypic landscape representing the “proliferation versus apoptosis” across different simulations. Color bars represent scores calculated as the values of “*proliferation*−*apoptosis*”. The bins in the figure are generated using the stat_summary_2d function from ggplot. (C) Schematic diagram of the computational functions for sensitivity and utility under multi-valued scenarios.

To demonstrate the potential of BioLogical, we applied this model and its dynamic framework to investigate additional logical behaviors beyond attractors. The multi-valued system load function can be used to input the data (Fig. 10A). The system state phenotype landscape can be analyzed by randomly sampling model states to identify potential stable states, which are then projected onto a principal component analysis (PCA) plot and colored according to their cell fate scores [37]. In multi-valued scenarios, the observed phenotypes inherently exhibit a wide range of variations due to their diverse discrete values—a feature that contrasts with Boolean logic models, which require multi-gene comprehensive scoring to construct the landscape. Fig. 10(B) shows that as the range of selectable values increases, the landscape becomes progressively clearer, with more detailed troughs emerging distinctly. The order parameters of logical paradigm in Boolean systems, such as sensitivity and effectiveness, can be effectively analyzed, as illustrated in Fig. 10(C).

**Fig. 11:**
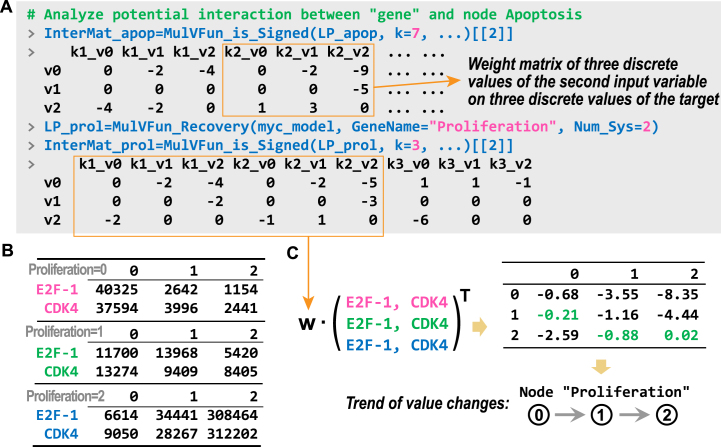
Analysis of interaction matrix among multi-valued variables and target nodes. (A) Schematic illustration of the interaction analysis between input and target nodes. (B) Distribution of input variable states. (C) Computation of value change trends.

Under a multi-valued framework, the trend of value changes in a node can be more precisely analyzed. Fig. 11(A) illustrates the “sign” function within multi-valued contexts, where the function outputs an interaction matrix. This matrix characterizes how various discrete values of each input variable influence specific target node states. The preferred trend of the target value is determined by the distribution of input variable values across stable states, obtained through random sampling (see the appendix for details). Moreover, the transition rule is determined by the highest score of $W{\vec{p}}_{k_{i},v_{j}}$. For instance, following a simulation, the distributions of the regulatory genes E2F-1 and CDK4 were statistically analyzed across different states of Proliferation (Fig. 11B). These distributions were normalized and multiplied by the weight matrix to derive state preference patterns, as illustrated in Fig. 11(C). The results indicate that, within the current system, Proliferation=2 represents the most favorable state, consistent with phenotype landscapes (Fig. 10B). However, not all nodes transition sequentially from 0→1→2; Apoptosis follows an alternative phenotypic pattern characterized by preference patterns 2→0 and 1→0.

## 5 Package performance

BioLogical is implemented entirely in C++ and typically achieves high computational efficiency. However, due to inherent limitations of current algorithms, certain functions may incur significant time costs, particularly as the scale of computation increases. We evaluated the computational performance of several key simulation and analysis functions, including DNS_CoreDyn, DNS_DamageSpread, MulVFun_Sensitivity, and MulVFun_Effectiveness. Benchmarks were conducted on a server equipped with an AMD EPYC 7763 processor (2.45 GHz) and 2 TB of RAM.

**Fig. 12:**
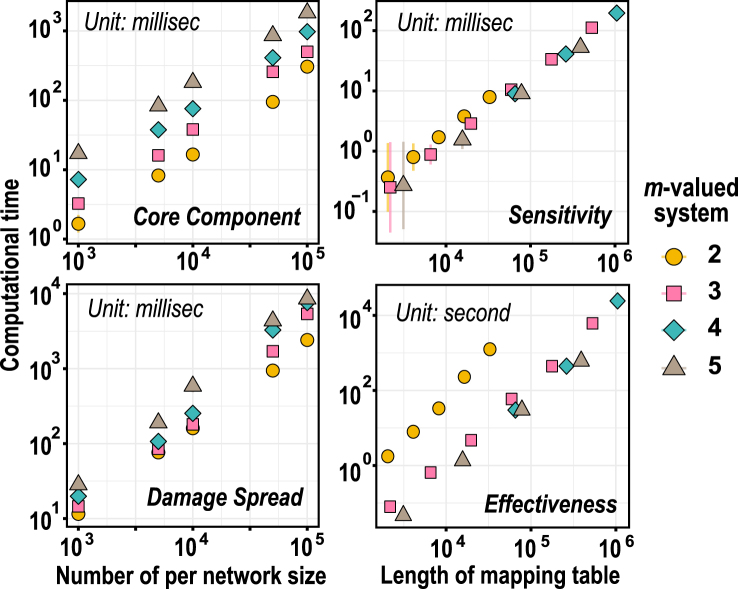
Performance evaluation of functions. The two functions on the left are implemented as Kauffman networks with an in-degree of 4 and system sizes of $10^{3}$, $5\times 10^{3}$, $10^{4}$, $5\times 10^{4}$, and $10^{5}$, respectively. The two functions on the right are configured with varying numbers of input parameters (k). Given the exponential growth of the mapping table ($∼m^{k}$), their table lengths are employed as the evaluation metric. The corresponding parameters are as follows: $k_{m=2}=11∼15$, $k_{m=3}=7∼12$, $k_{m=4}=8∼10$, and $k_{m=5}=5∼8$, which balances approximate table lengths, m values, and computational time. Note that the time units differ across the four figures, as shown in the upper-left corner of each figure. All simulations employ fully random logical paradigms to represent the most extreme scenarios, with the number of discrete values per node set to 2, 3, 4, and 5. For each parameter configuration, 1000 independent simulations are performed to calculate average performance.

Fig. 12 shows that, within a certain scale ($∼10^{5}$), the execution time of the system analysis function remains acceptable (left panels) and does not exhibit computational bottlenecks, growing linearly with increasing system size. For paradigm analysis functions, as the number of inputs (k) increases, the size of the mapping table grows exponentially, whereas computation time increases linearly with the length of the mapping table rather than with k. Sensitivity analysis is computationally efficient; however, determining the minimum disjunctive normal form of logical paradigms is NP-complete [38]. All functions involving the Quine-McCluskey method, such as *_QMForm, *_Complexity, *_Effectiveness, exhibit a significant increase in computation time, bounded above by a computational complexity of O($k^{2}m^{2k+1}$) [22]. The asterisk (*) denotes the Boolean and multi-valued functions in BioLogical, referred to as BoolFun and MulVFun, respectively. Memory consumption remains within acceptable limits given current hardware capabilities and increases linearly with both system size and mapping table length. The maximum memory consumption during the simulation was less than 550 MB, observed during the execution of the effectiveness analysis.

## 6 Discussion

Exploring the intrinsic logic of gene networks is essential for understanding the fundamental principles of life processes. Although simplified and abstracted from certain details, discrete models serve as powerful analytical tools, enabling systematic and computational interpretations of the universal behaviors exhibited by genetic interactions. From another perspective, the analysis of logical rules provides valuable insights into the design of synthetic gene circuits that mimic natural systems, as well as into the evolutionary patterns of gene regulatory networks. Thus, uncovering the inherent logical principles governing genetic interactions holds profound significance for both basic and applied biological research.

By integrating recent advances in algorithms with the growing availability of omics data, we developed the BioLogical R package, a comprehensive tool designed for the analysis of logical paradigms, system dynamics criteria, and order parameters. BioLogical offers extensive functionality for logical decomposition and the analysis of static and dynamic system properties, along with built-in gene network datasets to facilitate rapid exploratory analysis. Furthermore, all associated concepts have been extended to multi-valued systems to meet the complex and diverse requirements of analytical frameworks. The long-term objective is to establish a unified pipeline for logical model analysis. To this end, BioLogical provides hierarchical functional interfaces that support a broad range of applications, including interactive exploration, large-scale modeling, and advanced software development, making it particularly well-suited for the bioinformatics and computational biology communities. BioLogical still has room for improvement. The current version is primarily designed for top-down analysis and does not yet support the network inference in multi-omics contexts. The analytical capabilities for system attractors have not yet been integrated, and certain functions still require heuristic algorithms to further optimize their efficiency.

Overall, our package offers a comprehensive suite of foundational logical and dynamic analysis pipelines. The dynamic framework and functional modules are not only applicable to biosystems but also provide a robust foundation for analyzing other complex systems, serving as an infrastructure for future package development.

## 7 Appendix: mathematical concepts, definitions and algorithms

### 7.1 Canonical logical paradigms

**Canalization.** This paradigm emphasizes conditional outcomes within specific inputs. As shown in Fig. 4(A), the mapping result remains 0 when $x_{2}=1$, irrespective of the values of other variables. In this case, $x_{2}$ is termed the canalizing variable, the value “1” of $x_{2}$ is the canalizing value, and the corresponding value 0 of $f(\vec{x})$ is the canalized value associated with this canalizing variable. Hierarchical “canalizing-canalized” pairs, organized from higher to lower levels (e.g., $x_{2}$, $x_{5}$, $x_{3}$, $x_{1}$, $x_{4}$), constitute nested canalization structures [4], [39].

For a multi-valued system (denoted as M), canalizing and canalized values can be represented as subsets of M. Eq. (2) illustrates an example of a k-input canalization paradigm in M,

(2) $$f_{M}^{k}(\vec{x})=\left\{ \begin{array}{l} S_{v_{1}}^{o}\;\;x_{v_{1}}\in S_{v_{1}}^{i}\\ S_{v_{2}}^{o}\;\;x_{v_{1}}\notin S_{v_{1}}^{i},\;x_{v_{2}}\in S_{v_{2}}^{i}\\ \ldots \\ S_{v_{j}}^{o}\;\;x_{v_{1}}\notin S_{v_{1}}^{i},\;\ldots ,\;x_{v_{j-1}}\notin S_{v_{j-1}}^{i},\;x_{v_{j}}\in S_{v_{j}}^{i}\\ \mathrm{Other}\;\mathrm{Cases}. \end{array}\right.$$

Where the indices {$v_{1},v_{2},\ldots ,v_{j}$} form an ordered, non-repetitive sampling from {1,2,…,k}. The symbols $S_{v_{∼}}^{i}$, $S_{v_{∼}}^{o}$ denote the sets of canalizing and canalized values, respectively, at the layer of $x_{v_{∼}}$. These sets satisfy the constraints $\left|S_{v_{∼}}^{o}|⩽|S_{v_{∼}}^{i}|⩽|M\right|$ and series mapping relations $S_{v_{∼}}^{i}\mapsto S_{v_{∼}}^{o}$.

**Threshold, signed and monotonic patterns.** The meanings of these three concepts are similar. Typical threshold paradigms are represented as Fig. 4(E). The threshold for its mapping result is 0, depending on whether the constant term should be included in the threshold. Similarly, in the case of M, it is expressed as:

(3) $$f_{M}^{k}(\vec{x})=\Theta_{m}\left(\sum_{i}^{k}w_{i}x_{i}+\theta \right).$$

Where $w_{i}\in R$ represents the weight associated with k inputs; θ∈R is a constant value; and $\Theta_{m}$ denotes a step function with m intervals. The sum of all inputs and θ collectively determines the output [40].

Let ${\vec{x}}^{\left(i,j\right)}$ denote the input vector with $x_{i}=j$ (the meaning is the same in the following text). If $w_{i}>0$ and a<b mean that $f({\vec{x}}^{\left(i,a\right)})⩽f({\vec{x}}^{\left(i,b\right)})$ always holds, then the variable $x_{i}$ is regarded as having a consistently positive effect. The opposite case, $w_{i}<0$, is referred to as a consistently negative one. These positive/negative effects constitute *sign*-definite regulatory paradigms [13]. If all variables of $f(\vec{x})$ exhibit consistently positive effects, the paradigm is referred to as monotonically increasing; conversely, the case of consistently negative effects is referred to as monotonically decreasing [41].

**Dominant paradigms.** This type stresses the roles of majority or minority components in determining outputs, as applied in game theory [20], where states are incomparable. Our algorithms adopt the dominant component (majority) definition. These paradigms can be quantified through corresponding weights and baselines,

(4) $$f_{M}^{k}(\vec{x})=\arg\max_{s}\left\{\sum_{x_{i}=s}w_{i}+\theta_{s}\right\}.$$

Where $w_{i}$ denotes the weight of variable i; $\theta_{s}$ represents the baseline of the discrete state s. For instance, consider a 3-input binary dominant paradigm characterized by parameters $\left\{w_{1},w_{2},w_{3}\right\}$ and $\left\{\theta_{a},\theta_{b}\right\}$. The output for the input $\left\{x_{1}=a,x_{2}=b,x_{3}=a\right\}$ depends on the maximum of “$w_{1}+w_{3}+\theta_{a}$” and “$w_{2}+\theta_{b}$”. When considering interaction between any two states, Eq. (4) can be extended to more general forms,

(5) $$f_{M}^{k}(\vec{x})=\arg\max_{s}\left\{{\left[W^{\left(1\right)}W^{\left(2\right)}\;\ldots W^{\left(k\right)}\right]}_{\left(m\times mk\right)}I_{\left(mk\right)}+\vec{\theta }\right\}$$

Where **W**${\;}^{\left(i\right)}$ denotes the state interaction matrix of variable i. The element ($w_{ab}^{\left(i\right)}$) of matrix (**W**${\;}^{\left(i\right)}$) represents the influence of state $s_{b}$ on state $s_{a}$. I denotes an mk-dimensional indicator vector representing the discrete values of k variables. If the input vector $\vec{x}$ satisfies $x_{i}=s_{j}$, set the ((i−1)k+j)-th element of I to 1; otherwise, set it to 0. $\vec{\theta }$ represents the baselines as defined in Eq. (4). The matrix [**W**${\;}^{\left(i\right)}$] constitutes an interaction tensor, in which the interactions exhibit sign-definite properties, functioning as signed paradigms in multi-valued contexts.

### 7.2 Paradigm’s decomposition and conversion

**Logic decomposition.** The Quine-McCluskey method (QMCM) is a classical algorithm for deriving the minimal disjunctive normal forms (DNF) of Boolean expressions [21]. DNFs require all clauses to be connected solely by logical OR operators, with each clause comprising literals linked exclusively by logical AND operators. DNFs enable logical inference in pathways and support the design of genetic circuits.

The multi-valued extension of QMCM builds upon Pétrik’s framework, which defines multi-valued AND and OR operators ($\wedge_{M}$ and $\vee_{M}$) [22]. $\vee_{M}$ denotes the maximum value within a set, represented as ${\left\{\;\right\}}_{\max}$; accordingly, clauses are redefined as *assertions*, {r:C}. An assertion denotes a set of conditional vectors (C) that satisfy the corresponding results (r), namely $\forall \vec{x}\in C\subset M^{k}$ $\Rightarrow f_{M}^{k}(\vec{x})=r\in M$. C is equivalent to a k-dimensional vector, where the i-th component is ${\vec{x}}^{\left(i,s_{i}\right)}$ and $s_{i}\in \{M,*\}$ (“∗” represents a wildcard character). The multi-valued DNF can be expressed as follows,

(6) $$f_{M}^{k}(\vec{x})={\left\{\;\{r_{1}\text{:}C_{1}\},\;\{r_{2}\text{:}C_{2}\},\;\ldots ,\;\{r_{i}\text{:}C_{i}\}\;\right\}}_{\max}.$$

BioLogical enables the analysis of DNF in both Boolean and multi-valued scenarios. The QMCM also plays a fundamental role in functions related to edge effectiveness and logical complexity, etc.

**Polynomial conversion.** Any Boolean expression has an equivalent polynomial representation involving arithmetic operations [23]. The higher-order terms in these polynomial forms provide an alternative approach to quantifying the nonlinearity of paradigms. BioLogical employs the Boolean satisfiability solver Z3 [19], to convert Boolean or multi-valued logical paradigms into polynomial or high-order threshold paradigms, as illustrated in Fig. 4(D). In multi-valued systems, the product operator is defined as a modulo operation (${⊗}_{M}$), satisfying the properties $0{⊗}_{M}x=x$, $a{⊗}_{M}b=b{⊗}_{M}a$, $a{⊗}_{M}b=(a+b)\;\mathrm{mod}\;\;m$.

### 7.3 Order parameter

**Sensitivity (**S**).** This parameter quantifies the impact of input perturbations on the resulting mapping [12]. Its multi-valued definition can be represented as follows,

(7) $$S(f_{M}^{k}(\vec{x}))=m^{-k}\sum_{\vec{x}\in M^{k}}\sum_{i}\sum_{j}^{M\setminus \{x_{i}\}}\frac{f({\vec{x}}^{\left(i,x_{i}\right)}){⊗}_{e}f({\vec{x}}^{\left(i,j\right)})}{m-1}.$$

Where ${⊗}_{e}$ denotes the generalized Exclusive OR, defined such that $i{⊗}_{e}j=1$ if i=j, and $i{⊗}_{e}j=0$ if i≠j. The outer two summations enumerate all inputs ($M^{k}$) and variables (i), respectively, while the inner summation reflects the expected robustness of $x_{i}$ under perturbations.

**Effectiveness (**E**).** This parameter quantifies the extent to which each variable’s influence within logical paradigms contributes to results [8]. Based on multi-valued QMCM, the $E_{\text{input}}$ in multi-valued scenarios is defined as,

(8) $$E_{\text{input}}(f_{M}^{k}(\vec{x}))=k-m^{-k}\sum_{\vec{x}\in M^{n}}\sum_{s\in M}{\text{avg}}_{\vec{x}\in Q_{s}}(*).$$

Where $Q_{s}$ denotes all essential prime implicants that cover the subspace $\left\{\vec{x}|f_{M}^{k}(\vec{x})=s,\;\vec{x}\in M^{k}\right\}$. The notation ${\text{avg}}_{\vec{x}\in Q_{i}}(*)$ denotes the average over the invalid inputs within $\vec{x}$, where the symbol “∗” serves as a wildcard (consistent with its meaning in Eq. 6).

Variables exhibit different effective utility in influencing outputs, referred to as i-th edge effectiveness and defined as,

(9) $$E_{i}(f_{M}^{k}(\vec{x}))=m^{-k}\sum_{\vec{x}\in M^{k}}\sum_{s\in M}\text{Num}(x_{i}\neq *){|}_{\vec{x}\in Q_{s}}.$$

Where the notation $\text{Num}(x_{i}\neq *){|}_{\vec{x}\in Q_{s}}$ denotes the number over all $\vec{x}\in Q_{s}$ for which the i-th component $x_{i}$ is not a wildcard. Clearly, $E_{\text{input}}=\sum_{i}E_{i}(f_{M}^{k})$, where each $E_{i}(f_{M}^{k})\in [0,1]$. Correspondingly, $R_{i}(f_{M}^{k})\equiv 1-E_{i}(f_{M}^{k})$ denotes the input redundancy of the i-th variable. Please note that the states in analyzing E are incomparable; therefore, the essential prime implicants of each discrete state must be analyzed individually, rather than using the approach in Eq. (6).

**Prime implicants complexity (**C**).** The number of prime implicants in a paradigm provides a rough estimate of its logical complexity. The smaller values indicate fewer logical assertions and thus greater regularity. Regardless of whether the system is Boolean or multi-valued, complexity is defined as,

(10) $$C(f^{k})=\sum_{s\in M(B)}|Q_{s}|.$$

Where $Q_{s}$ is the same as in Eq. (8).

### 7.4 System analysis of GRN

**Benchmark of GRN.** Discrete dynamical systems are composed of nodes, links, and logical paradigms that collectively govern the system’s dynamic behavior and functionality. As canonical examples, gene regulatory networks (GRNs) typically exhibit stable or critical dynamics, driven by ordered logical interactions and topological structures [10], [11]. BioLogical includes three built-in GRN datasets for users to employ as benchmarks (Table 1).

**Table 1:** Genetic network sets.

Set name	Source and reference
CellCollective	70 networks from Cell Collective Web [42]
KadelkaSet	91 networks in Kadelka’s paper [43]
ThresholdModel	8 networks from [44], [45], [46], [47], [48], [49], [50], [51]

**Relevant components.** The algorithm comprises the following steps: *clamping nodes* and *pruning edges* [14]. The clamping node recursively searches all static nodes, either due to constant-value logical paradigms or because their inputs are fixed. Pruning edges entails the recursive identification of terminal nodes and invalid edges, which influence each other mutually. Terminal nodes are defined as those that do not regulate any other non-static nodes, while invalid edges are those that point exclusively toward static or terminal nodes. Generally, generative models contain numerous static nodes. However, in real GRNs, static nodes typically act as exogenous factors or controlled genes. The analysis of GRNs primarily involves the process of pruning edges.

**Dynamic core components.** Since BioLogical primarily focuses on the impact of logical paradigms on system behavior, the analysis of the dynamic core components is restricted to local interactions involving paradigms. The algorithm considers only the intermediary nodes in feedforward loops (FFLs), which take typical forms as shown in Fig. 6(B). Evaluating the logical paradigms and internal connections within FFLs can appropriately decouple FFLs into individual pathways. For instance, $f_{g_{2}}=g_{1}$ and $f_{g_{3}}=g_{1}\vee g_{2}$ indicate that node $g_{2}$ participates in redundant pathways rather than playing an essential role in the regulation of $g_{3}$. The formulaic representation is,

(11) $$\begin{array}{ll} \text{S} & \;=f_{\text{S}}(\vec{\text{P}},\text{I})=f_{\text{S}}(\vec{\text{P}},f_{\text{I}}(\vec{\text{P}}))\\ & \;=f_{\text{S}}^{\prime }(\vec{\text{P}}), \end{array}$$

where the new mapping relationship $f_{\text{S}}^{\prime }$ depends on the reduction of the logical correlation between $\vec{\text{P}}$ and I. In some cases, $f_{\text{S}}^{\prime }$ may not exist. This decoupling step depends on the process of logical paradigm optimization. Thus, it necessitates the iteration of clamping, pruning, and decoupling steps to determine the dynamic core components. The discarded nodes and irrelevant components are referred to as peripheral ones.

**Feedback loop (FBL) analysis.** The algorithm in BioLogical firstly identifies all strongly connected components (SCCs) within GRN. The results comprise two types: the TRUE SCC that indeed forms closed loops, and the intermediate nodes. Subsequently, the algorithm employs the Johnson method to identify all possible cycles, defined as complete signal transfers from a node back to itself. Note that no heuristic optimization algorithm is used in this package. As a result, computational time may increase significantly when an SCC is large or highly coupled. It is recommended to employ dynamic core components for a simplified analysis.

### 7.5 Dynamic simulation

**Update rules.** The dynamic simulation framework supports both synchronous and asynchronous rules [3], [52], allowing for a wide range of scenarios. The key difference between the two approaches is whether all nodes in the system are updated simultaneously or a single node is selected at random for update at each time step. Moreover, by focusing solely on state transitions rather than the individual steps, the algorithm also offers a fast asynchronous rule—specifically, the random selection of updatable nodes. This approach facilitates a more rapid convergence to a stable state.

**Derrida’s damage spread.** Let $M^{n}\{{\vec{f}}_{M},T_{n}\}$ denote an n-variable multi-valued network characterized by topological connections $T_{n}$ and a set of configured logical paradigms ${\vec{f}}_{M}\equiv \{f_{1},f_{2},\ldots ,f_{n}\}$. The normalized distance due to damage spread is defined as,

(12) $$D(M^{n}\{{\vec{f}}_{M},T_{n}\})=\frac{1}{n}\sum_{i}^{n}\Delta_{i}(t_{\infty }).$$

Where $\vec{\Delta }$ denotes the difference between a state and its perturbed one. $t_{\infty }$ represents a sufficiently simulated time, allowing perturbations to either dissipate or propagate. The algorithm is compatible with both Boolean and multi-valued systems.

**Percolation of stable components.** Observing percolation behavior necessitates the introduction of spatial constraints into $M^{n}\{{\vec{f}}_{M},T_{n}\}$. The function is to embed the system into three types of planar lattices: square, hexagonal, and triangular. In these cases, the interaction occurs only locally. Percolation here is defined as the presence of a stable cluster that spans the entire system. A stable cluster denotes a set of nodes that, beginning from an arbitrary initial state and evolving over a certain period, remain unchanged within a given observation window. Spanning refers to the existence of a continuous path through which a stable cluster connects one boundary of the system to the opposite boundary.

## CRediT authorship contribution statement

**Yuxiang Yao:** Writing – review & editing, Writing – original draft, Visualization, Supervision, Software, Methodology, Funding acquisition, Conceptualization. **Dong Liu:** Software, Investigation, Data curation. **Zheting Zhang:** Investigation, Data curation. **Chengchen Zhao:** Investigation, Funding acquisition, Data curation. **Duanqing Pei:** Writing – review & editing, Supervision, Project administration, Methodology, Funding acquisition, Conceptualization.

## Declaration of competing interest

The authors declare that they have no known competing financial interests or personal relationships that could have appeared to influence the work reported in this paper.

## Data Availability

All data are contained in the package. The package and all codes are available at https://github.com/YuxiangYao/BioLogical.

## Footnotes

1 The unique solution is relative to the simplest disjunctive normal form of a Boolean function [21]. Because the arithmetic computation must yield exactly 0 or 1, the order of the involved factors is typically large. However, in multi-valued scenarios, the feasible solution may not be unique and depends on the configuration of system attributes, such as comparability [22].
